# Supplementary material for: An effective and rapidly degradable disinfectant from disinfection byproducts
Source: Nat Commun. 2024 Jun 7;15:4888. doi: 10.1038/s41467-024-48752-w (PMC11161644; doi:10.1038/s41467-024-48752-w)
Supplement: Supplementary file 1 — Supplementary Information [file 41467_2024_48752_MOESM1_ESM.pdf]

Supplementary Information

An effective and rapidly degradable disinfectant from disinfection byproducts

Jiarui Han†, Wanxin Li†, Xiangru Zhang*

Department of Civil and Environmental Engineering, The Hong Kong University of Science and Technology, Hong Kong SAR, China

†These authors contributed equally: Jiarui Han, Wanxin Li.

*Corresponding author. *E-mail address*: xiangru@ust.hk (X. Zhang).

This PDF file includes:

Supplementary Note 1
Supplementary Tables 1–5
Supplementary Figs. 1–9

Supplementary Note 1. Chemicals and reagents

The standard compounds of para-chloro-meta-xylenol (PCMX) and halogenated disinfection byproducts (DBPs) were purchased and summarized as follows: PCMX (≥98%), 5-chlorosalicylic acid (98%), 5-bromosalicylic acid (90%), 5-iodosalicylic acid (98%), 2,4-dichlorophenol (99%), 2,4-dibromophenol (95%), 2,6-dichlorobenzoquinone (2,6-DCQ, 98%), and 2,5-DCQ (98%) were obtained from Sigma–Aldrich. 2,4-Diiodophenol (95%) and 2,5-diiodohydroquinone (97%) were obtained from AstaTech Inc. 2,5-Dichlorohydroquinone (98%), 2,6-dichlorohydroquinone (98%), and 2,5-dibromohydroquinone (97%) were obtained from International Laboratory USA. Acetonitrile, methanol, 2-propanol, methyl tert-butyl ether (MtBE), and other chemicals were at reagent grade or higher and were supplied by Sigma–Aldrich. Ultrapure water (18.2 MΩ·cm) was provided by a water purification system (Cascada I, Pall, USA). Seawater collected locally was filtered with a 0.45 μm filter, autoclaved at 121 °C for 30 min, and cooled to room temperature. The pretreated seawater was used for polychaete cultivation, developmental toxicity bioassay, and degradation experiments.

Supplementary Table 1 | *E. coli* inactivation rate constants and the doses of PCMX and the nine DBPs for achieving 3-log inactivation of *E. coli* (pH 7.2, contact time 5 min).

**Compound**	***k*** **(L mg $^{-1}$ h^{-1})**	**Dose (mg L^{-1})**
PCMX	0.66	188
2,4-Dichlorophenol	0.30	472
2,4-Dibromophenol	0.28	341
2,4-Diiodophenol	0.79	112
2,5-Dichlorohydroquinone	0.05	946
2,5-Dibromohydroquinone	0.03	1450
2,5-Diiodohydroquinone	0.13	459
5-Chlorosalicylic acid	<0.01	/
5-Bromosalicylic acid	<0.01	/
5-Iodosalicylic acid	<0.01	/

Supplementary Table 2 | Degradation of PCMX and the DBPs in seawater with and without solar irradiation.

**Compound**	**With solar irradiation**			**Without solar irradiation**		
	***k* (h^{-1})**	**R^2**	**Half-life (h)**	***k* (h^{-1})**	**R^2**	**Half-life (h)**
PCMX	2.70×10^{-3}	0.992	257	/	/	/
2,4-Dichlorophenol	1.54×10^{-2}	0.999	45	/	/	/
2,4-Dibromophenol	2.01×10^{-2}	0.999	35	/	/	/
2,4-Diiodophenol	8.30×10^{-3}	0.989	83	/	/	/
2,5-Dichlorohydroquinone	9.28×10^{-2}	0.978	0.12	8.85×10^{-2}	0.972	0.13
2,5-Dibromohydroquinone	7.19×10^{-2}	0.988	0.16	7.85×10^{-2}	0.978	0.15
2,5-Diiodohydroquinone	7.26×10^{-2}	0.976	0.16	7.26×10^{-2}	0.975	0.16

Supplementary Table 3 | *E. coli* inactivation by 2,5-DCQ and 2,6-DCQ (pH 7.2, contact time 5 min).

**Compound**	**Dose (mg L^{-1})**	**Log reduction (Mean ± SD)**
2,5-DCQ	15	3.78 ± 0.40
	7.5	0.77 ± 0.14
2,6-DCQ	15	4.24 ± 0.22
	7.5	0.76 ± 0.16

Supplementary Table 4 | Pathogen inactivation rate constants and required doses of 2,6-DCQ and PCMX for achieving 3-log inactivation of *E. coli*, *S. aureus*, *C. albicans* and MS2 (pH 7.2, contact time 5 min).

Pathogen	Compound	k (L mg^{-1} h^{-1})	Dose (mg L^{-1})	R^2
E. coli	2,6-DCQ	5.50	12.8	0.998
	PCMX	0.66	188	0.983
S. aureus	2,6-DCQ	1.21	28.6	0.991
	PCMX	0.22	308	0.964
C. albicans	2,6-DCQ	1.88	35.4	0.927
	PCMX	0.22	320	0.951
MS2	2,6-DCQ	0.85	46.1	0.969
	PCMX	0.036	1010	0.946

Supplementary Table 5 | Degradation kinetics of 2,6-DCQ with or without solar irradiation.

	**pH**	***k*** **(h^{-1})**	**Half-life (h)**	**R^2**
With solar irradiation	6.2	0.168	4.13	0.998
	7.2	0.217	3.20	0.975
	8.2	0.520	1.33	0.990
Without solar irradiation	6.2	0.009	76.2	0.963
	7.2	0.046	13.4	0.967
	8.2	0.397	1.74	0.990

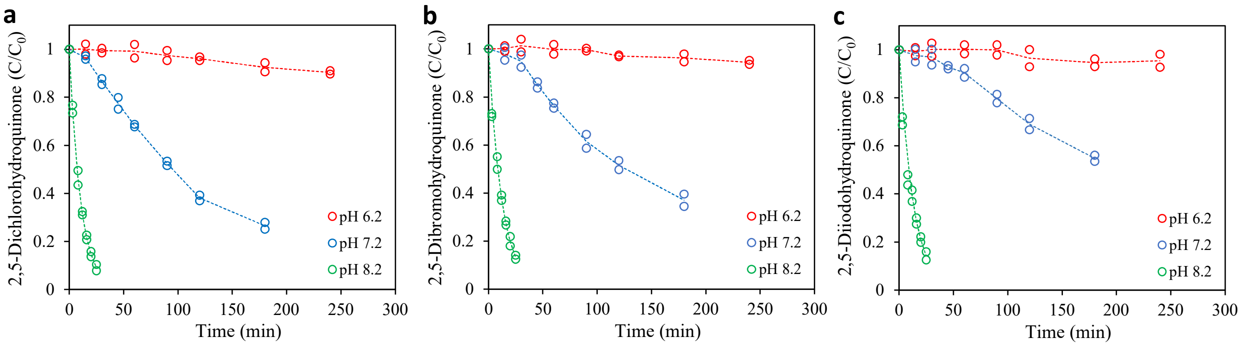

Supplementary Fig. 1 | Effect of pH on the degradation of (a) 2,5-dichlorohydroquione, (b) 2,5-dibromohydroquinone, and (c) 2,5-diiodohydroquinone without solar irradiation. The data presented were from two independent experiments. Source data are provided as a Source Data file.

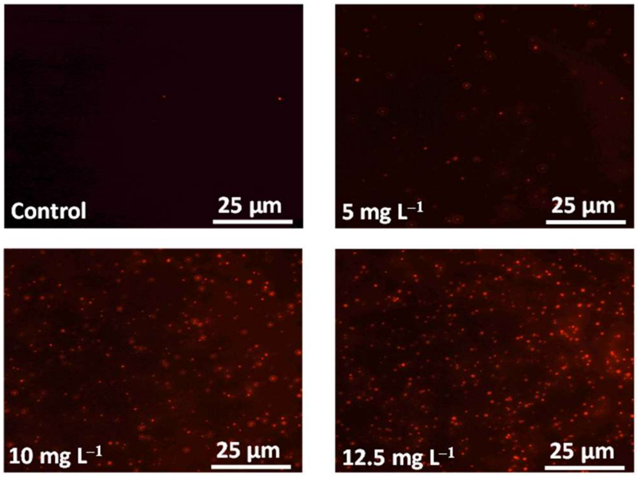

Supplementary Fig. 2 | Fluorescence microscope images of *E. coli* with treatment of 0, 5, 10, 12.5 mg L^{-1} of 2,6-DCQ (pH 7.2, contact time 5 min).

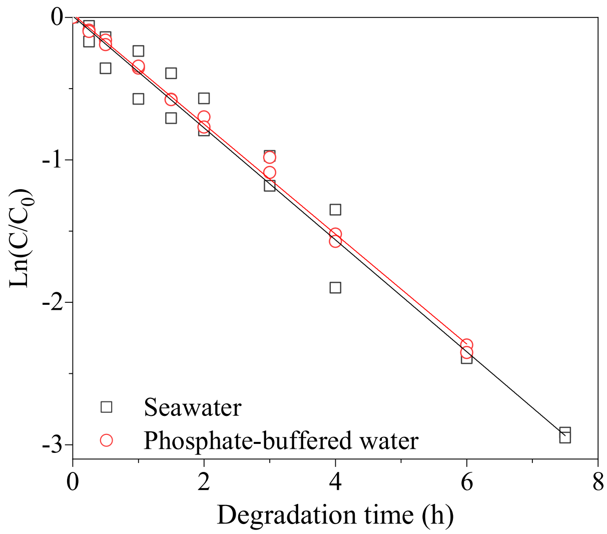

Supplementary Fig. 3 | Degradation of 2,6-DCQ at pH 8.2 in seawater and phosphate-buffered water in darkness. The data presented were from two independent experiments. Source data are provided as a Source Data file.

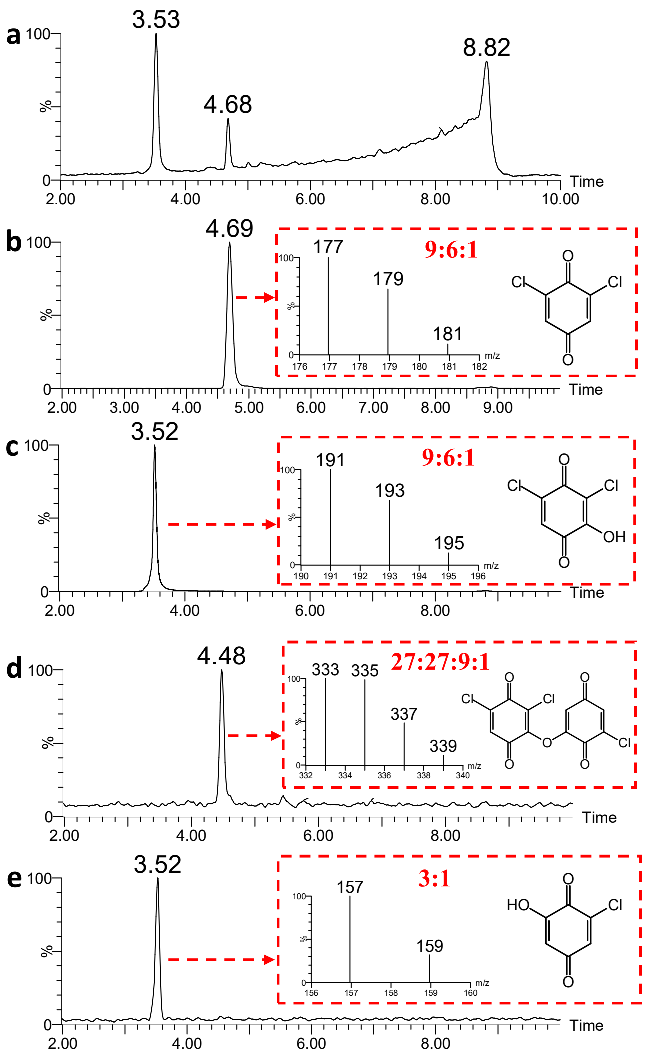

Supplementary Fig. 4 | (a) Ultra performance liquid chromatography/electrospray ionization-triple quadrupole mass spectrometry (UPLC/ESI-tqMS) full scan chromatogram of the 2,6-DCQ solution after 5 hours of degradation in darkness. UPLC/ESI-tqMS multiple reaction monitoring chromatograms and spectra of (b) 2,6-DCQ, (c) 3-hydroxyl-2,6-dichloro-1,4-benzoquinone (OH-DCQ), (d) dimeric product via OH-DCQ coupling with 2,6-DCQ, and (e) 3-hydroxyl-6-monochloro-1,4-benzoquinone.

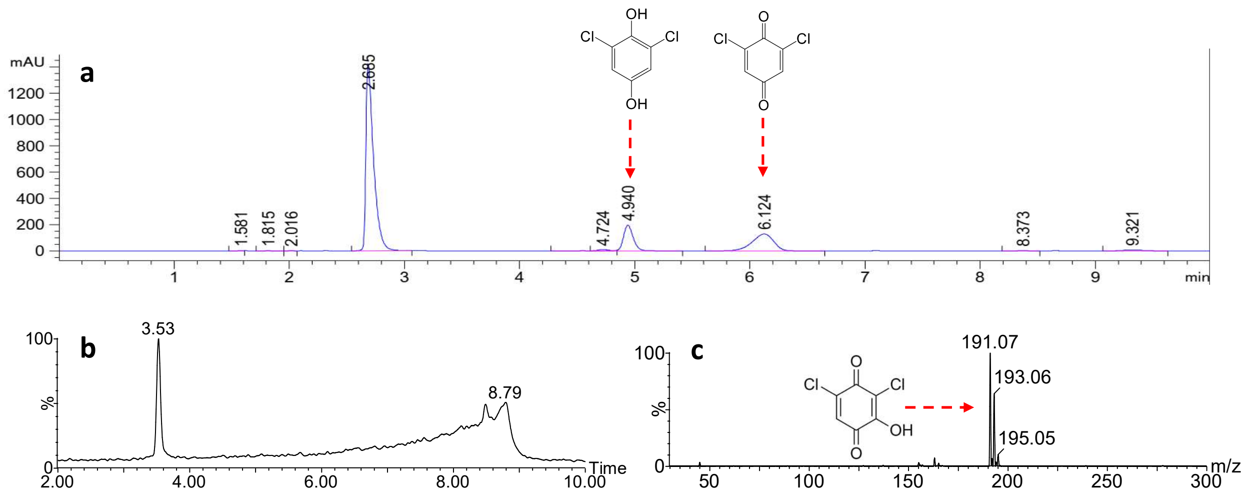

Supplementary Fig. 5 | (a) High performance liquid chromatography (HPLC) chromatogram (280 nm) of the 2,6-DCQ solution at a degradation time of 72 hours, (b) UPLC/ESI-tqMS full scan chromatogram of the collected fraction, and (c) the corresponding mass spectrum at the retention time of 3.53 min.

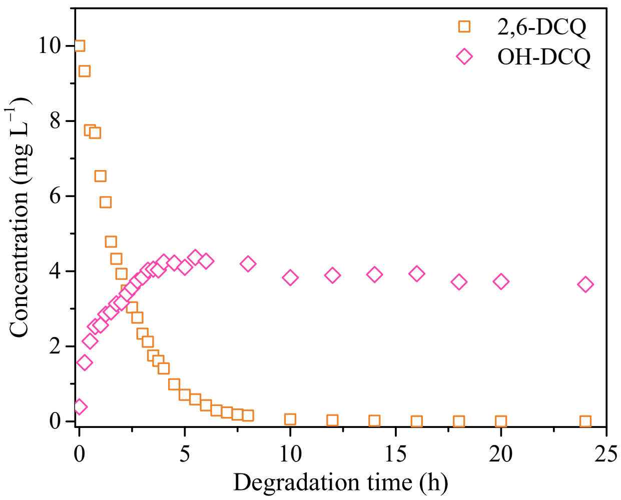

Supplementary Fig. 6 | Concentrations of 2,6-DCQ and OH-DCQ with degradation time. Source data are provided as a Source Data file.

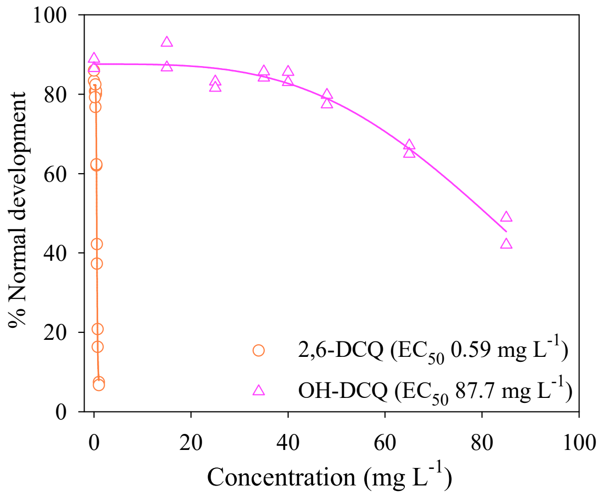

Supplementary Fig. 7 | Comparative developmental toxicity of 2,6-DCQ and OH-DCQ to *P. dumerilii* embryos (150 embryos per sample). The data presented were from two independent experiments. Source data are provided as a Source Data file.

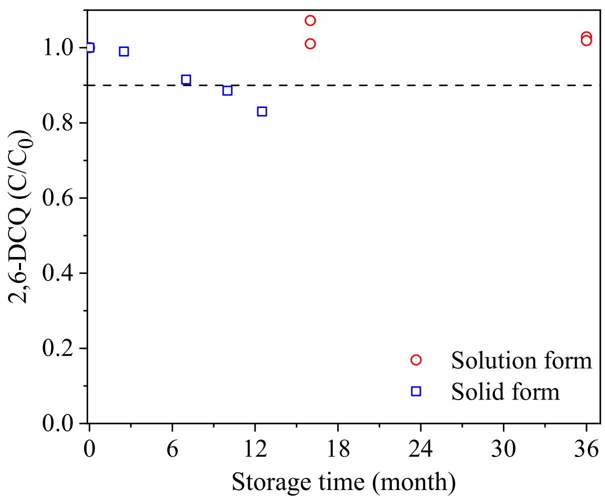

Supplementary Fig. 8 | The concentrations of 2,6-DCQ in solution form (using 2-propanol as the solvent) and solid form at different storage times. Source data are provided as a Source Data file.

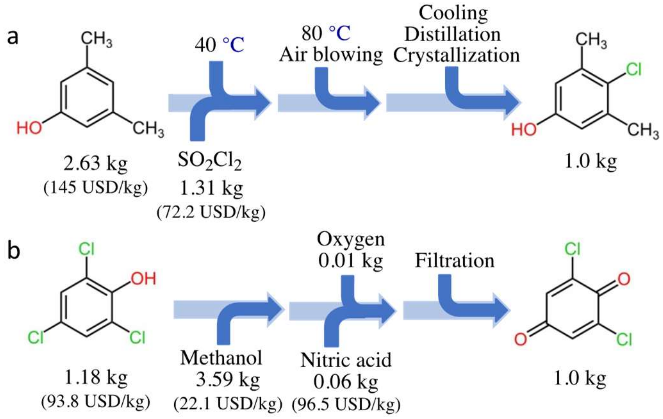

Supplementary Fig. 9 | Synthesis flowcharts of (a) PCMX and (b) 2,6-DCQ. The unit prices of raw materials were obtained from a global supplier Alfa Aesar (https://alfaaesar.com/).
