# Supplementary material for: An effective and rapidly degradable disinfectant from disinfection byproducts
Source: Nat Commun. 2024 Jun 7;15:4888. doi: 10.1038/s41467-024-48752-w (PMC11161644; doi:10.1038/s41467-024-48752-w)
Supplement: Supplementary file 3 — Reporting Summary [file 41467_2024_48752_MOESM3_ESM.pdf]

nature portfolio

Corresponding author(s): Xiangru Zhang

Last updated by author(s): Apr 30, 2024

Reporting Summary

Nature Portfolio wishes to improve the reproducibility of the work that we publish. This form provides structure for consistency and transparency in reporting. For further information on Nature Portfolio policies, see our Editorial Policies and the Editorial Policy Checklist.

Statistics

For all statistical analyses, confirm that the following items are present in the figure legend, table legend, main text, or Methods section.

n/a	Confirmed	
☐	☒	The exact sample size (*n*) for each experimental group/condition, given as a discrete number and unit of measurement
☐	☒	A statement on whether measurements were taken from distinct samples or whether the same sample was measured repeatedly
☒	☐	The statistical test(s) used AND whether they are one- or two-sided<br>*Only common tests should be described solely by name; describe more complex techniques in the Methods section.*
☒	☐	A description of all covariates tested
☒	☐	A description of any assumptions or corrections, such as tests of normality and adjustment for multiple comparisons
☐	☒	A full description of the statistical parameters including central tendency (e.g. means) or other basic estimates (e.g. regression coefficient) AND variation (e.g. standard deviation) or associated estimates of uncertainty (e.g. confidence intervals)
☒	☐	For null hypothesis testing, the test statistic (e.g. *F*, *t*, *r*) with confidence intervals, effect sizes, degrees of freedom and *P* value noted<br>*Give P values as exact values whenever suitable.*
☒	☐	For Bayesian analysis, information on the choice of priors and Markov chain Monte Carlo settings
☒	☐	For hierarchical and complex designs, identification of the appropriate level for tests and full reporting of outcomes
☒	☐	Estimates of effect sizes (e.g. Cohen's *d*, Pearson's *r*), indicating how they were calculated

Our web collection on statistics for biologists contains articles on many of the points above.

Software and code

Policy information about availability of computer code

Data collection	No software was used.
Data analysis	Origin 2021, SigmaPlot 12, ImageJ and Microsoft Excel were used for data analysis.

For manuscripts utilizing custom algorithms or software that are central to the research but not yet described in published literature, software must be made available to editors and reviewers. We strongly encourage code deposition in a community repository (e.g. GitHub). See the Nature Portfolio guidelines for submitting code & software for further information.

Data

Policy information about availability of data

All manuscripts must include a data availability statement. This statement should provide the following information, where applicable:

- Accession codes, unique identifiers, or web links for publicly available datasets
- A description of any restrictions on data availability
- For clinical datasets or third party data, please ensure that the statement adheres to our policy

Source data are provided with this paper as a Source data file.

Research involving human participants, their data, or biological material

Policy information about studies with human participants or human data. See also policy information about sex, gender (identity/presentation), and sexual orientation and race, ethnicity and racism.

Reporting on sex and gender	Not applicable
Reporting on race, ethnicity, or other socially relevant groupings	Not applicable
Population characteristics	Not applicable
Recruitment	Not applicable
Ethics oversight	Not applicable

Note that full information on the approval of the study protocol must also be provided in the manuscript.

Field-specific reporting

Please select the one below that is the best fit for your research. If you are not sure, read the appropriate sections before making your selection.

☐ Life sciences ☐ Behavioural & social sciences ☒ Ecological, evolutionary & environmental sciences

For a reference copy of the document with all sections, see nature.com/documents/nr-reporting-summary-flat.pdf

Ecological, evolutionary & environmental sciences study design

All studies must disclose on these points even when the disclosure is negative.

Study description	This study aimed to identify an effective and rapidly degradable/detoxified disinfectant from disinfection byproducts (DBPs). The experimental units include antimicrobial activity tests, degradation tests, and toxicity tests, using a factorial design structure. Duplicate experiments were conducted for each test. For the antimicrobial activity tests, treatment factors included the types of DBPs (n = 10), the species of microbes (n = 4), DBP concentrations (n = 4 or 5) and contact time, and pH values (n = 3). For the degradation tests, treatment factors included the types of DBPs (n = 10), pH values (n = 3), and with/without sunlight. For the toxicity tests, treatment factors included the types of DBPs (n = 7) and degradation times (n = 1–8).
Research sample	The research samples include (1) four microbes for broad-spectrum antimicrobial testing and (2) 10 halophenolic DBPs. The four microbes include Escherichia coli (gram-negative bacteria), Staphylococcus aureus (gram-positive bacteria), Candida albicans (fungi), and bacteriophage MS2 (viruses). The microbes were obtained from the American Type Culture Collection. The 10 DBPs were selected based on the authors' previous work on the toxicity and photodegradation of DBPs.
Sampling strategy	Not applicable.
Data collection	The authors recorded the data of different experiments.
Timing and spatial scale	The experiments and data collection started in Jan 2022 and ended in Feb 2024.
Data exclusions	No data were excluded.
Reproducibility	All the experiments were conducted in duplicate. Repeated experiments were successful.
Randomization	The DBPs were grouped based on their chemical structural characteristics. The microbes were grouped based on their Morphological and physiological characteristics.
Blinding	Blinding was used during data acquisition as one author conducted the experiments and another author conducted data acquisition.

Did the study involve field work? ☐ Yes ☒ No

Reporting for specific materials, systems and methods

We require information from authors about some types of materials, experimental systems and methods used in many studies. Here, indicate whether each material, system or method listed is relevant to your study. If you are not sure if a list item applies to your research, read the appropriate section before selecting a response.

Materials & experimental systems

n/a	Involved in the study
☒	☐ Antibodies
☒	☐ Eukaryotic cell lines
☒	☐ Palaeontology and archaeology
☐	☒ Animals and other organisms
☒	☐ Clinical data
☒	☐ Dual use research of concern
☒	☐ Plants

Methods

n/a	Involved in the study
☒	☐ ChIP-seq
☒	☐ Flow cytometry
☒	☐ MRI-based neuroimaging

Animals and other research organisms

Policy information about studies involving animals; ARRIVE guidelines recommended for reporting animal research, and Sex and Gender in Research

Laboratory animals	This study involved the embryos of a marine polychaete Platynereis dumerilii.
Wild animals	This study did not involve wild animals.
Reporting on sex	This information was not collected.
Field-collected samples	This study did not involve samples collected from the field.
Ethics oversight	*Identify the organization(s) that approved or provided guidance on the study protocol, OR state that no ethical approval or guidance was required and explain why not.*

Note that full information on the approval of the study protocol must also be provided in the manuscript.

Plants

Seed stocks	This study did not involve plants.
Novel plant genotypes	Not applicable.
Authentication	Not applicable.
